# A new subgingival sling suture technique for periodontally accelerated osteogenic orthodontics

**DOI:** 10.1097/MD.0000000000030601

**Published:** 2022-09-16

**Authors:** Shuining Wang, Chang Wen, Sihong Li, Junli Zhu, Jingjing Shu, Dong Yang

**Affiliations:** a The State Key Laboratory Breeding Base of Basic Science of Stomatology (Hubei-MOST) & Key Laboratory of Oral Biomedicine Ministry of Education, School and Hospital of Stomatology, Wuhan University, Wuhan, China; b School and Hospital of Stomatology, Wuhan University, Wuhan, China.

**Keywords:** bone augmentation, gingiva recession, guided bone regeneration, modified sling suture, periodontally accelerated osteogenic orthodontics

## Abstract

This study aimed to design a modified subgingival sling suture for periodontally accelerated osteogenic orthodontics (PAOO) as well as evaluate postoperative effects including gingival recession (GR), alveolar bone crest resorption, dental plague accumulation on sutures and alveolar bone augmentation. Twelve patients with bone defects in the anterior alveolar region of the mandible were included in this study. Subgingival sling suture, developed from traditional sling suture, was applied in modified PAOO operation. Probing depth, bleeding index, and GR were assessed, and cone-beam computerized tomography and laser microscope for thread surface were evaluated at baseline, postoperative 1 and 3 months to analyze the effects. Alveolar bone thickness on the labial side at the midpoint of the middle third of the root increased from 0.96 ± 0.28 mm to 3.38 ± 0.61 mm (*P* < .01), and that of the apical third advanced from 1.26 ± 0.33 mm to 3.61 ± 1.02 mm (*P* < .01), both exhibiting significant increase. No significant alveolar bone crest loss, probing depth increase, GR, and attachment loss was observed. This modified PAOO operation, associated with novel subgingival sling suture, productively augments alveolar bone volume and addresses problems in terms of GR and vertical loss of alveolar bone.

## 1. Introduction

Bone fenestration and dehiscence on the buccal aspect of alveolar bone present a high natural incidence rate in the population as local contributing factors to periodontitis.^[1,2]^ Meanwhile, periodontology and orthodontics are closely associated in clinical practice. In conventional orthodontic treatments, differentiation of periodontal tissues occurs when mechanical force was exerted on the periodontium, and the treatment lasted 2 years or more on average.^[3]^ For patients with Class II and Class III malocclusion, movement of the anterior mandibular teeth toward the labial aspect usually issues in labial bone resorption in the corresponding area, sometimes even fenestration and dehiscence which expose the root directly. All these above can imperil alveolar bone volume and induce gingiva recession.^[4–6]^ Attention must be paid to a more effective and secure orthodontic therapeutic approach by both periodontists and orthodontists.

In 2001, Wilcko et al^[7–9]^ introduced a novel technique named “periodontally accelerated osteogenic orthodontics (PAOO)” for the first time. PAOO, a periodontal surgical operation method with favorable therapeutic outcomes, has been proven to possess the characteristics of facilitating tooth movement and augmenting bone plate thickness around the root.^[8]^ This innovative technique incorporates exposure of the alveolar bone via flap reflection, linear decortications in the inter-radicular space, solitary perforations in the alveolar bone over the radicular surface, and grafting evenly in areas that have undergone corticotomy. PAOO, along with its theoretical basis and practical application, inaugurated new methods and mindsets for orthodontic treatment.^[8,10]^

Due to various techniques of the procedure, many problems there remain unsolved in conventional PAOO. Bo et al^[11]^ reported a striking vertical alveolar bone loss in the anterior mandibular teeth region after the PAOO operation during the follow-up. Likewise, a separate study by Coscia et al^[12]^ spotted a remarkable reduction in both thickness and height of the alveolar bone crest during decompensation in patients with Class III malocclusion under treatment. Apart from alveolar bone crest resorption, postoperative gingiva recession appears as a common issue of PAOO. Brugnami et al^[13]^ discovered in the PAOO follow-up that gingiva recession can be induced by marginal bone loss due to operation. Similarly, Kreisler et al^[14]^ reported gingiva atrophy to the tune of 0.6 mm in some PAOO cases and assumed that it was attributable to sulcular incision together with flap reflection.

To address these problems, we ameliorated the conventional PAOO technique in the anterior area by placing an incision 3 mm away from the marginal gingiva and devising a novel subgingival method for sling suture. This suture technique ensures that threads are covered by gingiva and minimizes dental plaque accumulation via diminishing exposure of threads. This study aimed to evaluate the postoperative outcomes and ultimately look forward to its effects on plaque accumulation, productively facilitating bone augmentation and gingival recession (GR).

## 2. Materials and Methods

### 2.1. Study participants

Twelve patients diagnosed with dental malocclusion with loss of bone plate thickness in the anterior alveolar region from September 2020 to December 2021 were included in this study. The procedures were approved by the Ethics Committees of School and Hospital of Stomatology Wuhan University, and all patients signed an informed consent agreement for this study.

The inclusion criteria were as follows: aged between 18 and 30 (including 18 and 30); good oral hygiene; cone-beam computerized tomography (CBCT) results confirming thinness (<1.5 mm) of labial bone plate in the lower anterior alveolar region or/and dehiscences or fenestrations on the labial surface; no systemic diseases; no active periodontitis and no more than 1 mm of probing depth (PD); no root anomaly and malformation, no pulpitis and apical periodontitis; no history of smoking.

Patients with one or more of the following characteristics were excluded: periodontium anomaly, attachment loss; labial bone thickness more than 1.5 mm, more than 1 mm of PD; less than 2 mm of the width of keratinized gingiva; mucogingival anomaly; pregnant or lactating; administration of any medication that affects bone metabolism; loss to follow-up.

### 2.2. Surgical procedure

#### 2.2.1. Flap reflection.

**Figure 1.**
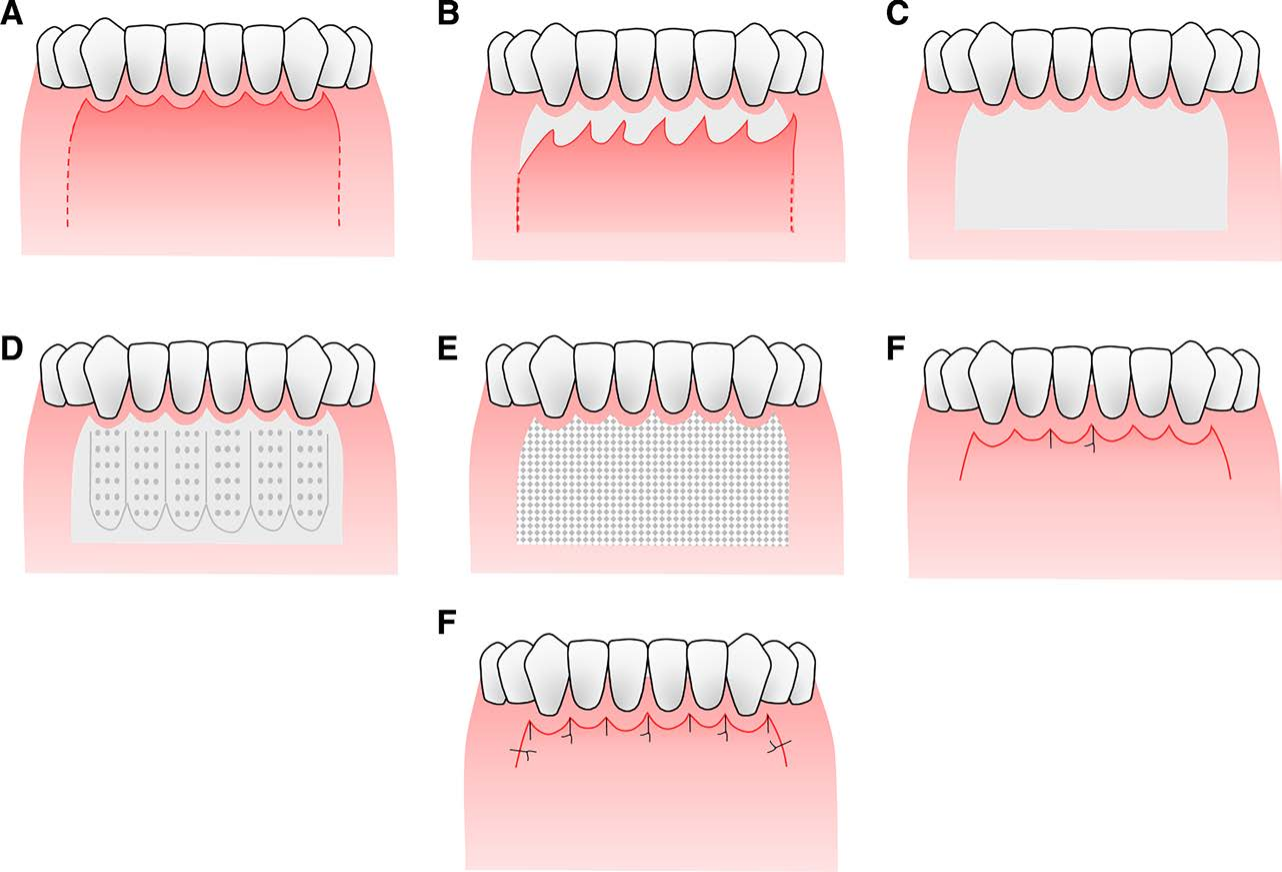
Modified PAOO operation. (A) A scalloped incision was adapted to protect the alveolar crest and prevent gingival recession; (B) flap reflection; (C) exposure of labial alveolar bone in the surgical area; (D) based on CBCT results, vertical corticotomies were positioned in the inter-radicular area and then connected with horizontal corticotomies placed 2 mm beyond the root apex, punctate decortications were subsequently performed; (E) bone graft materials was placed on recipient sites; (F) flap reposition and subgingival sling suture; (G) completed sutures. CBCT = cone-beam computerized tomography, PAOO = periodontally accelerated osteogenic orthodontics.

A scalloped incision below the gingival margin was adapted for the purposes of preserving soft tissue nearby,^[8,15]^ protecting the alveolar bone crest and thus preventing postoperative GR in the surgical area (Fig. 1). Incision was placed 3 mm below the anterior gingival margin, flanked by two vertical releasing incisions extending 5 mm (Fig. 1A). Then periosteotomy was performed and the flap was reflected 2 mm beyond the apices of the anterior teeth to create a full-thickness mucoperiosteal flap. Subsequently, a partial-thickness flap separating the periosteum flap from the overlying mucosal layer (Figs. 1B and 3A) was made to release the pressure of the flap (Figs. 1C and 3B).

**Figure 2.**
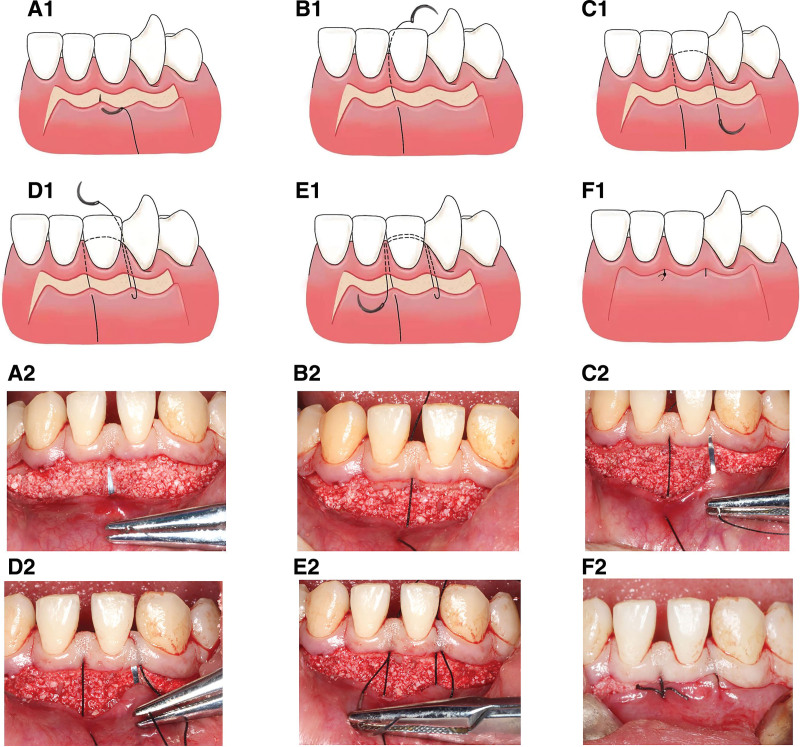
Modified subgingival sling suture technique for PAOO. (A1 and A2) The suture needle penetrates the papilla of the free flap; (B1 and B2) the needle is inserted below the buccal papilla and extracted from the gingival sulcus on the lingual aspect; (C1 and C2) the needle penetrates the adjacent interdental papilla and the outer-surface of the initial flap; (D1 and D2) the suture is reinserted below the second papilla; (E1 and E2) the suture is brought round the tooth lingually and allowed to exit the initial papilla buccally; tie the knot; (F1 and F2) merely limited segments of suture are exposed. PAOO = periodontally accelerated osteogenic orthodontics.

#### 2.2.2. Corticotomy.

**Figure 3.**
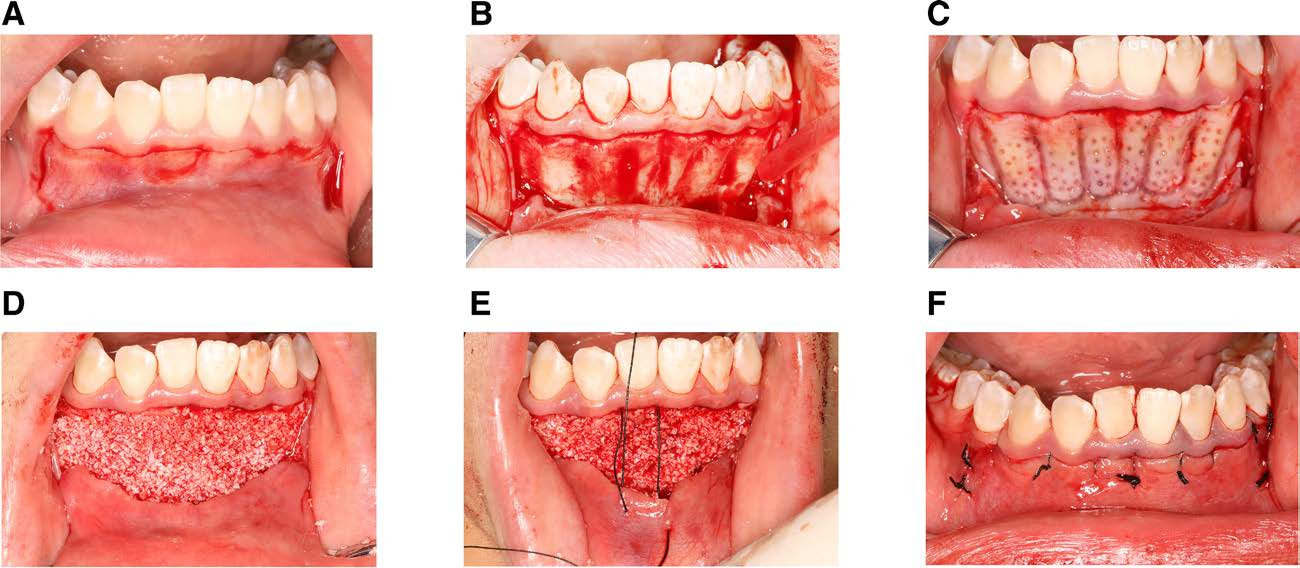
PAOO operation with modified sling suture. (A) Scalloped incision; (B) flap reflection and exposure of buccal alveolar bone; (C) corticotomies and punctate decortications; (D) bone graft material was placed on operation area; (E and F) intraoral photograph after completed sutures. Merely limited segments at both ends of the suture were exposed and the bulk of it was covered subgingivally. PAOO = periodontally accelerated osteogenic orthodontics.

Based on the morphology and length of the root obtained by using CBCT (NewtomVG, Verona, Italy), corticotomies were performed using a round bur or a piezoelectric surgical device (diameter less than or equal to 1 mm). Vertical corticotomies were in the inter-radicular area, extending 2 mm beyond the apices of the teeth, and then were connected with horizontal corticotomies apically (Figs. 1D and 3C). Punctate decortications are recommended to place between vertical grooves if the alveolar bone is of sufficient thickness, while they are omitted under the circumstance of thin bone (Figs. 1D and 3C).

#### 2.2.3. Grafting.

Artificial bone graft material (Bio-Oss^®^; Geistlieh AG, Wolhusen, Switzerland) which was mixed with blood collected from the surgical area was positioned on the recipient sites (an estimated amount of 0.75–1.5 g) without compaction (Figs. 1E and 3D).

#### 2.2.4. New subgingival sling suture technique.

A novel subgingival sling suture technique was devised by our team. The flap was repositioned. The suture needle penetrates the papilla of the free flap (Fig. 2A1 and A2). Then it is inserted below the buccal gingival papilla and extracted from the gingival sulcus on the lingual aspect (Fig. 2B1 and B2), encircling the tooth lingually in the gingival sulcus before it penetrates the adjacent interdental papilla and then the outer surface of the initial free flap (Fig. 2C1 and C2). The suture is then reinserted below the second papilla (Fig. 2D1 and D2), brought round the tooth lingually, and allowed to exit the initial papilla buccally (Fig. 2E1 and E2). Eventually, the knot is tied and the modified sling suture is completed (Fig. 2F1 and F2). This technique ensures the exposure of the suture to the oral environment is minimal because it merely exposes limited segments at both ends of the suture and the bulk of it was covered subgingivally (Fig. 1F). Suturing was performed with 4-0 nonabsorbable polyester and a simple surgeon’s knot was placed. The additional releasing incisions on both sides were sutured with regular interrupted sutures (Figs. 1G and 3F).

#### 2.2.5. Postoperative management.

The patient was instructed to apply fibroblast growth factor to the surgical area for a week after surgery to facilitate wound healing and use oral rinses for hygiene maintenance. Anti-inflammatory treatment of antibiotics was administered 3 days after surgery and the stitches were removed a week later. All patients were submitted to CBCT examination to appraise the effect of bone grafts in PAOO.

### 2.3. Evaluations of new subgingival sling suture technique

#### 2.3.1. Clinical assessments.

The following periodontal clinical parameters were measured at baseline, 1- and 3-month after surgery: GR: the distance from the cemento-enamel junction to the gingival margin; PD: the probing distance from the gingival margin to the bottom of the gingival sulcus; bleeding index: A blunt probe was inserted into the gingival sulcus or bottom of the periodontal pocket, and the degree of bleeding were observed after 30 seconds.

#### 2.3.2. Analysis of exposed and hidden sutures.

Using this new subgingival sling suture technique, sutures can be divided into two segments: the exposed section where sutures are above the gingiva and the hidden section where sutures are covered under the gingiva. The plaque often accumulates on stitches exposed to the oral cavity due to daily food chewing. On the contrary, the hidden subgingival sutures are not susceptible to plaque. Before removing the sutures, methylene blue was used to stain the exposed parts. Afterward, the sutures were removed and the length of the exposed and the hidden segments were measured respectively.

#### 2.3.3. Evaluations of surface morphology.

Given that the surface morphology of the thread denotes its ability to retain plague, assessments on the surface morphology (ISO25178) were conducted. We performed surface profile analysis using laser microscope VK-X Profiler (Keyence, Itasca, IL) on the acquired surface morphology and surface roughness of the exposed and hidden subgingival segments. Surface morphology was analyzed by the root mean square gradient of the profile. Surface roughness was evaluated via the sum of its contour lines of peak material volume (*V*_mp_).

#### 2.3.4. Radiographic evaluation by CBCT.

**Figure 4.**
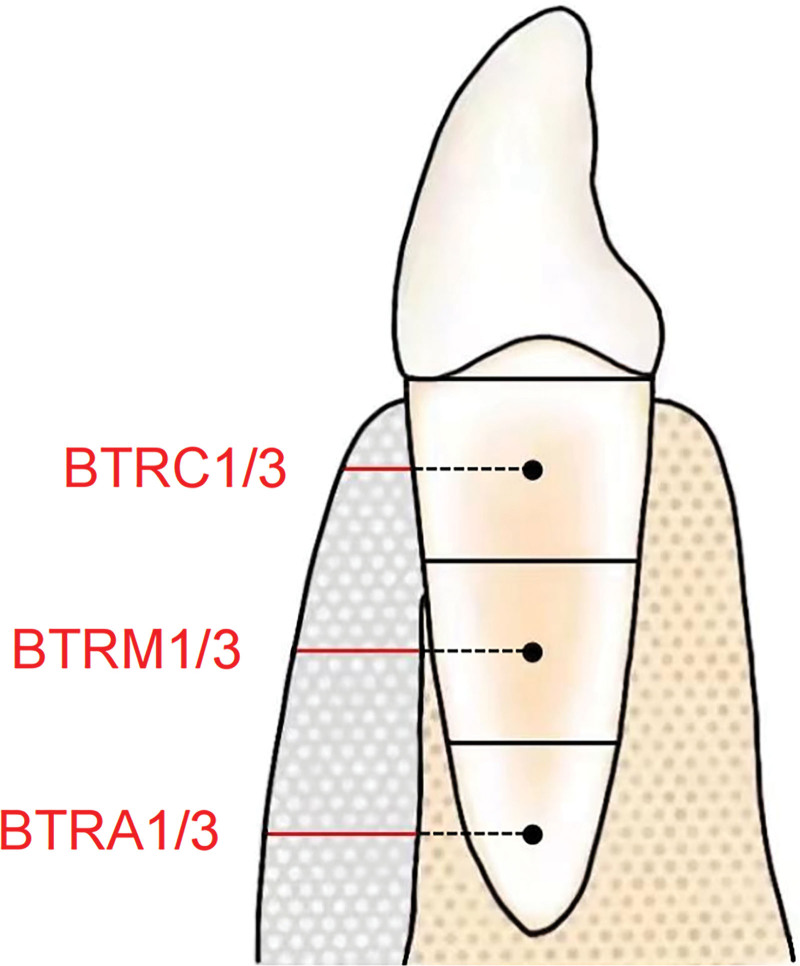
Evaluation of bone thickness augmentation after osseous grafting in PAOO. Changes of BTRC1/3, BTRM1/3 and BTRA1/3 were quantified and compared (preoperative vs postoperative, colored by red). BTRA1/3 = bone thickness of root apical 1/3, BTRC1/3 = bone thickness of root coronal 1/3, BTRM1/3 = bone thickness of root middle 1/3, PAOO = periodontally accelerated osteogenic orthodontics.

The DICOM data were imported to CT’s analysis software and the volume maps were reconstructed. A lingual-buccal section was made on the axis of each root of the lower anterior teeth and the distance from the root surface to the bone surface (Fig. 4) was determined in millimeters (mm). Changes (preoperative vs postoperative) of the bone thickness of root coronal 1/3, bone thickness of root middle 1/3, and bone thickness of root apical 1/3 were quantified and compared. The measurement was repeated by the same person one week later. The average of the two measurements is shown as the final result.

### 2.4. Statistical analysis

All collected data were analyzed by statistical software SPSS v22 (IBM, Armonk, NY) and was presented as mean (standard deviation). The Friedman test was employed to compare different time points of before-and-after surgery. The least significant difference multiple-comparison post hoc test was used after the Friedman test. Statistically significant differences were set as the value of .05 (*P* < .05).

## 3. Results

### 3.1. Results of clinical parameters

**Figure 5.**
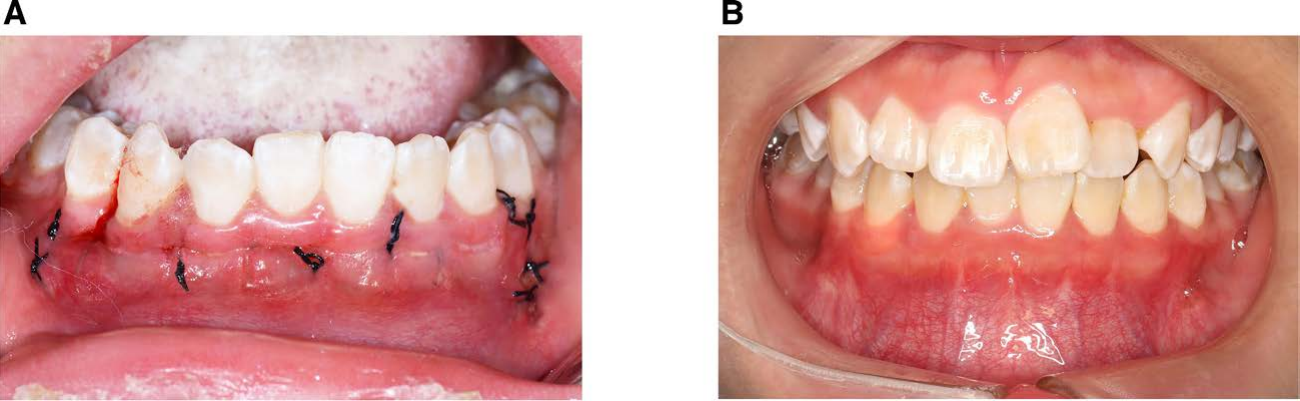
Photographs after PAOO surgery. (A) Materia alba can be observed on sutures in the operation area at 2 weeks after PAOO surgery; (B) gingiva recovered at 3 months after PAOO surgery. PAOO = periodontally accelerated osteogenic orthodontics.

Like other regular periodontal surgeries, its postoperative reactions including swelling and pain subsided within a week after surgery. Records of periodontal clinical parameters started from 1 month after the surgery in consideration of the unsuitability for probing within 4 weeks after periodontal surgery. As shown in Table 1, no GR occurred at baseline, 1 and 3 months after surgery, indicating that soft tissue attachment level remain unchanged. Similarly, PD showed no significant difference among the 3-time points: 0.62 mm preoperative, 0.66 mm 1 month, and 0.64 mm 3 months. Bleeding index significantly increased at 1 month and then significantly reduced at 3 months after PAOO surgery, showing that gingiva appeared hyperemic or inflammatory to a limited extent after this procedure (Fig. 5A) and recovered to normal condition after 3 months (Fig. 5B).

**Table 1:** Periodontal parameters over time (mm).

Parameters	BL	1M	3M	Mulitplecomparison†		
				BL-1M	1M-3M	BL-3M
PD	0.62 (0.12)	0.66 (0.13)	0.64 (0.12)	0.433	0.683	0.694
BI	0.71 (0.27)	1.93 (0.47)	0.69 (0.26)	<0.0001*	<0.0001*	0.886
GR	0.16 (0.03)	0.16 (0.02)	0.15 (0.03)	0.999	0.370	0.372

Data are presented as mean (standard deviation).

1M = 1 month after surgery, 3M = 3 months after surgery, BI = bleeding index, BL = baseline, GR = gingival recession, PD = probing depth.

* Significant difference, *P* < .01.

† Multiple comparison, least significant difference multiple-comparison post hoc test.

### 3.2. Results of the analysis on exposed and hidden sutures

**Figure 6.**
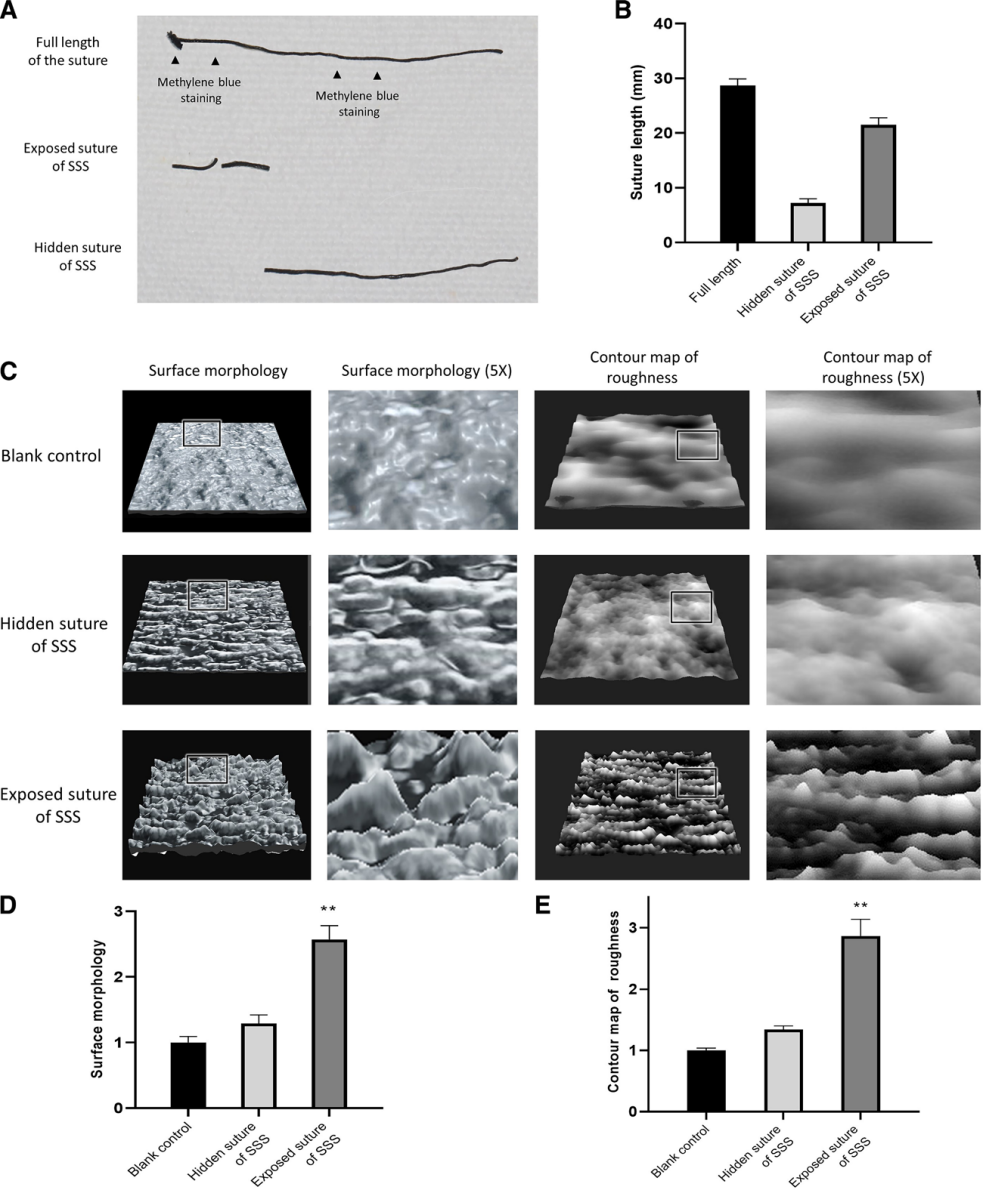
Laser analysis on surface profile of the exposed and non-exposed area. (A) Methylene blue was used to stain the exposed suture (arrows) and then the suture was severed according to staining; (B) statistically there is significant difference between the length of the exposed sutyre and the total; (C–E) according to surface morphology analysis by laser microscope, normal sutures, as blank control, represent smooth surface and shows significantly low *V*_mp_ variation; there suggested no significant difference in surface roughness between hidden suture and blank control; exposed sutures showed irregularity and notable variation in surface morphology with substantial pore structures, and its *V*_mp_ suggested statistic difference compared with blank control (*P* < .05). PAOO = periodontally accelerated osteogenic orthodontics, SSS = subgingival sing suture. ** Significant difference, *P* < .05.

The exposed section consisted of two segments, with a total length of 6.97 mm accounting for 23.7% of the whole suture. While the hidden sections reached 22.47 mm and took up 76.3% of the whole suture, which represented a statistical difference in comparison with the length of exposed sections (Fig. 6A and B).

According to surface morphology analysis by laser microscope (Fig. 6C–E), normal sutures, as blank control, represent smooth surface and its contour map of roughness shows significantly low *V*_mp_ variation. Though hidden sutures exhibited rough surfaces in terms of morphology and the root mean square gradient result suggested no significant difference in surface roughness between the hidden sutures and blank control. In contrast, exposed sutures showed irregularity and notable variation in surface morphology with substantial pore structures. There existed a distinct difference in surface roughness and a multitude of peaks and troughs on the contour map. *V*_mp_ suggested a statistical difference compared with blank control (*P* < .05).

### 3.3. CBCT showed significant alveolar bone augmentation buccally

**Figure 7.**
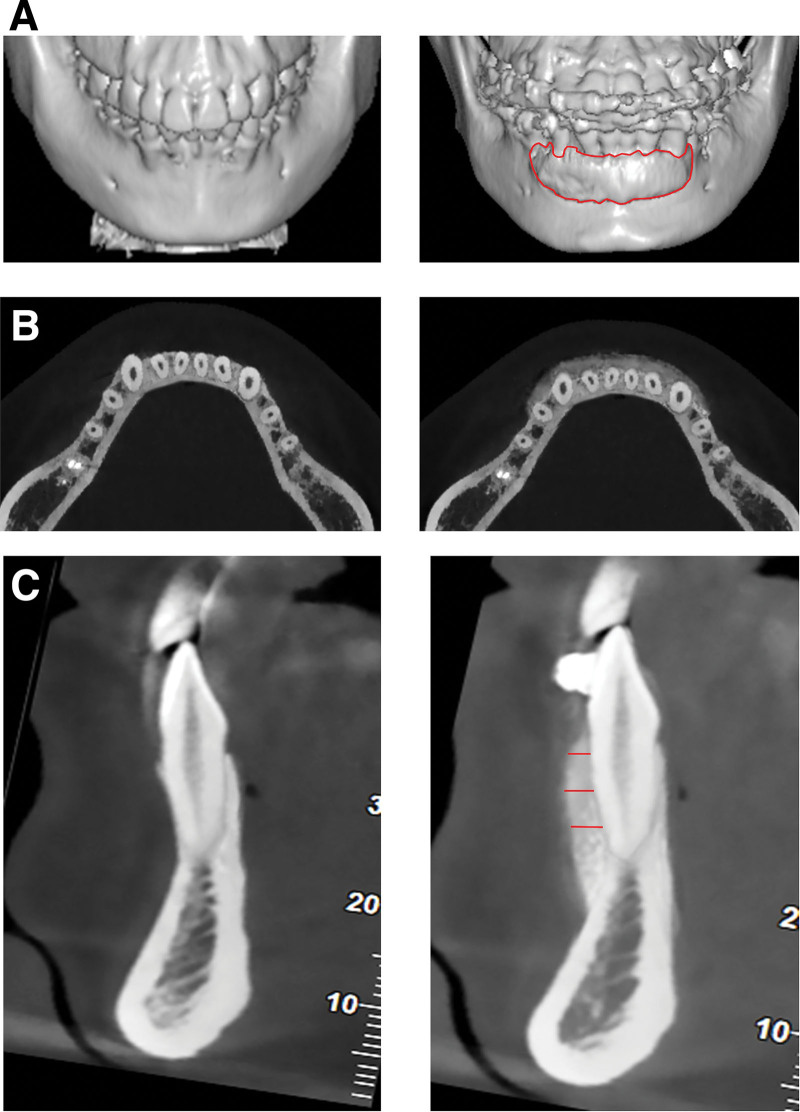
Preoperative and postoperative CBCT images. (A) Image-based 3-dimensional reconstruction before surgery and at postoperative 3-month; area of bone augmentation was colored by red; (B) CBCT images of horizontal view shows significant alveolar bone augmentation in the same mandibular anterior area at postoperative 3-month; (C) images of buccal-lingual view shows significant alveolar bone augmentation in the buccal radicular surface after PAOO; red lines show BTRC1/3, BTRM1/3 and BTRA1/3. BTRA1/3 = bone thickness of root apical 1/3, BTRC1/3 = bone thickness of root coronal 1/3, BTRM1/3 = bone thickness of root middle 1/3, CBCT = cone-beam computerized tomography, PAOO = periodontally accelerated osteogenic orthodontics.

CBCT results (before and 3 months after treatment) are shown in Figure 7 and Table 2. The alveolar bone thickness on the labial side at the middle level of the root coronal third was 0.83 ± 0.17 mm before surgery, which leaped to 1.26 ± 0.31 mm after modified PAOO surgery. Given treatment, that of the root middle third hiked from 0.96 ± 0.28 mm to 3.38 ± 0.61 mm, exhibiting statistical significance (*P* < .0001). Similarly, that of the root apical third advanced from 1.26 ± 0.33 mm to 3.61 ± 1.02 mm after the surgery, which indicated a boost in bone volume (*P* < .0001).

**Table 2:** Measurements of the variables for alveolar bone thickness (mm).

Parameters	BL	3M	*P* value
BTRC1/3	0.83 (0.17)	1.26 (0.31)	.0012*
BTRM1/3	0.96 (0.28)	3.38 (0.61)	<.0001*
BTRA1/3	1.26 (0.33)	3.61 (1.02)	<.0001*

Data are presented as mean (standard deviation).

3M = 3 months after surgery, BL = baseline, BTRC1/3 = bone thickness of root coronal 1/3, BTRM1/3 = bone thickness of root middle 1/3, BTRA1/3 = bone thickness of root apical 1/3.

* Significant difference, *P* < .01.

## 4. Discussion

This study for the first time designed a new subgingival sling suture technique and a modified PAOO procedure and evaluated their effects. Unlike the conventional technique of incision and suture, this new method is characterized by a scalloped incision 3 mm away from the gingival margin preserving the collar of gingival tissue and a modified sling suture. It inhibits postoperative GR and ensures esthetics in the anterior region concerning the gingival papilla and gingival margin, thereby remaining alveolar bone height as well as facilitating its osteogenesis effects.

Reverse overbite, open bite, deep overbite, dental crowding, accelerating canine movement after extraction of premolar, and other obstacles often occur in the practices of orthodontics,^[4,16]^ which considerably influence oral function and esthetics along with treatment design and time. Previous studies have demonstrated that the labial bone plate is more susceptible to resorption than the lingual bone plate.^[17]^ Thus studies on labial alveolar bone and labial gingiva figures much more prominently in the development of PAOO. Postoperative alveolar bone resorption is a common phenomenon in early PAOO procedures resulting from sulcular incision and the mucoperiosteal flap that cause full bone exposure.^[18]^ Kreisler et al^[14]^ reported that GRs up to 0.6 mm were observed in some cases performing sulcular incisions. Hence, many researchers initiated modified techniques to tackle these drawbacks of traditional PAOO. In 2009, Dibart et al^[19]^ originated Pizocision, a flapless corticotomy that combined traditional PAOO technique with a minimally invasive approach. This method adopted vertical gingival incision and dispensed with horizontal incision, inserting the head of the piezotome in the gingival openings and decorticating the alveolar bone to the depth of 3 mm. Canals on the bone surface are formed with vertical incisions linked. This approach is minimally invasive and beneficial for wound healing,^[20]^ whereas it entails special equipment and mastery of clinical skills, let alone that it is a blind operation. It is worth noting that the PAOO treatment was first proposed as “corticotomy” by Köle^[21]^ in the absence of “accelerated” in its definition. Owing to the discovery of a localized osteoporosis state in further experiments, a healing event called regional acceleratory phenomenon where the injury accelerated the normal regional healing processes,^[22]^ “corticotomy” was then altered to “PAOO”. Therefore, this standard name is continually adapted in this study while exploring the PAOO technique and suture method is the primary aim of our study. The result of accelerated osteogenesis remains to be explored and discussed in the future study.

This study put forward the application of scalloped incision leaving the collar of gingival tissue in PAOO. The incision devised in this study is grounded on the rationale that the biological width keeps approximately 2 mm^[23]^ and the depth of the gingival sulcus stays under 1 mm,^[24]^ altogether 3 mm. Placing the incision where the collar of gingival tissue is preserved instead of inside the gingival sulcus can protect the gingival margin and alveolar bone crest, prevent GR and vertical alveolar resorption, hence successfully address the two major issues of traditional PAOO.

The modified sling suture method innovated in this subject is particularly suitable for scalloped incision or its analogs. In conventional PAOO with interrupted interdental suture, a large area of flap reflection, flap contraction, and tension in suture generated by fillings of bone-grafting material inevitably, torn gingiva caused by interrupted suture and therefore loose stitches.^[25]^ Distinct from traditional sling suture, which expose the whole thread to the oral environment up to 24.9 mm, modified sling suture only allows very limited sections at both ends of the sutures exposed to the outside while the rest was covered. The length of the exposed suture, merely 6.97 mm, was reduced by 76.3%, diminishing the retention of food residue and plaque. Additionally, the suture is unaffected by tension and impact of food for its structural stability. Thus, this technique promises favorable conditions for healing. Notably, this method developed by our team has not been reported yet worldwide, and its long-term effects are being further observed. In addition, analyses were focused on the before-and-after measurements of set parameters, which exhibited statistically differences. Therefore, the modified technique is preliminary proved to be effective and feasible, while its superiority to conventional techniques remains to be investigated and discussed in the future study.

During this procure, it is recommended to use a round bur with a diameter of no more than 1 mm for grooving and punctate osteotomy, and compaction on bone substitutes should be avoided during grafting. Although there exists no reference to the advisable width for grooving and punctate corticotomy in previous studies on PAOO, an experiment on fracture healing in long bones by Johner^[26]^ and an examination of experimental bone defects by Schenk et al^[27]^ indicated the threshold for a primary osseous bridging is 1 mm. Similarly, Harris et al^[28]^ confirmed in experiments on dog hip bones that a single “jump” of the bone growth cannot exceed 1 mm. Blood clots of high quality play a crucial role in bone formation because of the potential for calcification into osseous tissue. Furthermore, bone grafting materials should be geometrical porous to facilitate the growth of blood vessels along fibers and particles, which is the osteo-conducive potential in osteogenesis. To achieve this purpose, it is critical to prevent bone materials from tight compaction. Additionally, it is broadly acknowledged that CBCT is an effective approach to observe osteogenesis in vitro without second surgery. Although biopsy is the gold standard of the maturity of bone tissue, it will cause additional injuries and is against the participants’ will.

There remain some deficiencies in this study. The results indicated remarkable augmentation in horizontal bone thickness at the level of the middle and apical third, however, that of the coronal third only slightly ascended. We attributed the disparity to our novel surgical procedure, in which the scalloped incision leaving the collar of gingival tissue effectively preserves the soft tissue of the gingival margin and papilla, hence resulting in inadequate bone augmentation in the coronal 1/4 to 1/3 region. Accordingly, this technique is not applicable for large bone defects strictly confined to the root coronal third, which unavoidably is the universal limitation with respect to all kinds of incisions preserving gingival papilla. Coincidentally this holdback also emerges in operations of conventional crevicular incision plus full-thickness flap reflection. Wang et al^[11]^ and Lee et al^[29]^ reported that CBCT results showed that bone augmentation in the root apical area surpasses that in the root coronal area. We theorized that it is the gravity and lip movement causing displacement of bone grafts towards the middle root and root apex.

Advantages of this modified procedure and modified sling suture include being easy to master, needlessness of sophisticated skills, being unrequired for special devices or ultrasonic bone scalpel, relatively healing fast, avoidance of materials displacement, morphological protection of gingival margin as well as alveolar bone, excellent short-term osteogenesis effect, and so on. The optimal amount of bong graft to maintain alveolar stability and its long-term effect remains to be further assessed.

This modified operation not only employs a scalloped incision to preserve the soft tissue of the gingival margin and papilla, hence precluding GR, but also maintains vertical alveolar bone height and avoids exposure of alveolar bone which prevents bone loss. Subgingival sling suture is designed to secure the stability of the suture and attenuate the irritations from plaque and contamination of Materia alba, therefore fostering bone healing.

## Acknowledgment

The authors thank Yi Guo for assistance with statistical analysis.

## Author contributions

**Conceptualization:** Dong Yang, Chang Wen, Shuining Wang.

**Data curation:** Shuining Wang, Sihong Li, Junli Zhu, Jingjing Shu.

**Funding acquisition:** Dong Yang.

**Investigation:** Dong Yang, Shuining Wang, Sihong Li, Junli Zhu, Jingjing Shu.

**Methodology:** Dong Yang, Chang Wen.

**Resources:** Shuining Wang, Dong Yang.

**Supervision:** Dong Yang.

**Writing – original draft:** Shuining Wang, Dong Yang.

## References

[1] Handelman CS. The anterior alveolus: its importance in limiting orthodontic treatment and its influence on the occurrence of iatrogenic sequelae. Angle Orthod. 1996;66:95–109; discussion 109–110. 8712499 10.1043/0003-3219(1996)066<0095:TAAIII>2.3.CO;2

[2] Leung CC Palomo L Griffith R. Accuracy and reliability of cone-beam computed tomography for measuring alveolar bone height and detecting bony dehiscences and fenestrations. Am J Orthod Dentofacial Orthop. 2010;137(4 Suppl):S109–19. 20381751 10.1016/j.ajodo.2009.07.013

[3] Ong MMA Wang H-L. Periodontic and orthodontic treatment in adults. Am J Orthod Dentofacial Orthop. 2002;122:420–8. 12411890 10.1067/mod.2002.126597

[4] Nimigean VR Nimigean V Bencze MA. Alveolar bone dehiscences and fenestrations: an anatomical study and review. Rom J Morphol Embryol. 2009;50:391–7. 19690764

[5] Wehrbein H Bauer W Diedrich P. Mandibular incisors alveolar bone, and symphysis after orthodontic treatment. A retrospective study. Am J Orthod Dentofacial Orthop. 1996;110:239–46. 8814023 10.1016/s0889-5406(96)80006-0

[6] Evangelista K de Faria Vasconcelos K Bumann A. Dehiscence and fenestration in patients with Class I and Class II Division 1 malocclusion assessed with cone-beam computed tomography. Am J Orthod Dentofacial Orthop. 2010;138:133.e131–7; discussion 133–5. 10.1016/j.ajodo.2010.02.021 20691344

[7] Wilcko MT Wilcko WM Bissada NF. An evidence-based analysis of periodontally accelerated orthodontic and osteogenic techniques: a synthesis of scientific perspectives. Semin Orthod. 2008;14:305–16.

[8] Murphy KG Wilcko MT Wilcko WM. Periodontal accelerated osteogenic orthodontics: a description of the surgical technique. J Oral Maxillofac Surg. 2009;67:2160–6. 19761909 10.1016/j.joms.2009.04.124

[9] Wilcko WM Wilcko T Bouquot JE. Rapid orthodontics with alveolar reshaping: two case reports of decrowding. Int J Periodontics Restorative Dent. 2001;21:9–19. 11829041

[10] Ma Z Zheng J Yang C. A new modified bone grafting technique for periodontally accelerated osteogenic orthodontics. Medicine. 2018;97:e12047. 30212935 10.1097/MD.0000000000012047 PMC6156025

[11] Bo W Shen G Fang B. Augmented corticotomy-assisted presurgical orthodontics of class III malocclusions: a cephalometric and cone-beam computed tomography study. J Craniofac Surg. 2013;24:1886–90. 24220368 10.1097/SCS.0b013e3182a245b3

[12] Coscia G Coscia V Peluso V. Augmented corticotomy combined with accelerated orthodontic forces in class III orthognathic patients: morphologic aspects of the mandibular anterior ridge with cone-beam computed tomography. J Oral Maxillofac Surg. 2013;71:1760.e1761–9. 10.1016/j.joms.2013.04.022 23773424

[13] Brugnami F Caiazzo A Mehra P. Can corticotomy (with or without bone grafting) expand the limits of safe orthodontic therapy? J Oral Biol Craniofac Res. 2018;8:1–6. 29556455 10.1016/j.jobcr.2017.11.001 PMC5854557

[14] Kreisler M Khl S Gockel R. Clinical evaluation of a modified marginal sulcular incision technique in endodontic surgery. Oral Surg Oral Med Oral Pathol Oral Radiol Endod. 2009;108:e22–28. 10.1016/j.tripleo.2009.08.005 19913716

[15] Lee EA. Aesthetic crown lengthening: classification, biologic rationale, and treatment planning considerations. Pract Proced Aesthet Dent. 2004;16:769. 15739921

[16] Kim Y Park JU Kook YA. Alveolar bone loss around incisors in surgical skeletal Class III patients. Angle Orthod. 2009;79:676–82. 19537864 10.2319/070308-341.1

[17] Yared K Zenobio EG Pacheco W. Periodontal status of mandibular central incisors after orthodontic proclination in adults. Am J Orthod Dentofacial Orthop. 2006;130:6.e1–8. 10.1016/j.ajodo.2006.01.015 16849063

[18] Düker J. Experimental animal research into segmental alveolar movement after corticotomy. J Maxillofac Surg. 1975;3:81–4. 1055773 10.1016/s0301-0503(75)80022-1

[19] Dibart S Sebaoun JD Surmenian J. Piezocision: a minimally invasive, periodontally accelerated orthodontic tooth movement procedure. Compend Contin Educ Dent. 2009;30:342–4, 346, 348–50. 19715011

[20] Milano F Dibart S Montesani L. Computer-guided surgery using the Piezocision technique. Int J Periodontics Restorative Dent. 2014;34:523–9. 25006769 10.11607/prd.1741

[21] Kole H. Surgical operations on the alveolar ridge to correct occlusal abnormalities. Oral Surg Oral Med Oral Pathol. 1959;12:515–9. 13644913 10.1016/0030-4220(59)90153-7

[22] Schilling T Müller M Minne HW. Influence of inflammation-mediated osteopenia on the regional acceleratory phenomenon and the systemic acceleratory phenomenon during healing of a bone defect in the rat. Calcif Tissue Int. 1998;63:160–6. 9685523 10.1007/s002239900508

[23] Carvalho BAS Duarte CAB Silva JF. Clinical and radiographic evaluation of the Periodontium with biologic width invasion. BMC Oral Health. 2020;20:116. 32299404 10.1186/s12903-020-01101-x PMC7164352

[24] Renvert S Persson GR. A systematic review on the use of residual probing depth, bleeding on probing and furcation status following initial periodontal therapy to predict further attachment and tooth loss. J Clin Periodontol. 2002;29(Suppl 3):82–9; discussion 90–1. 12787209 10.1034/j.1600-051x.29.s-3.2.x

[25] Liu X Fan B Abdelrehem A Ma Z. Membrane fixation for osseous graft stabilization in periodontally accelerated osteogenic orthodontics: a comparative study. BMC Oral Health. 2020;20:22. 31992277 10.1186/s12903-019-0964-5 PMC6988277

[26] Johner R. Zur Knochenheilung in Abhangigkeit von der Defektgrosse. Helv Chir Acta. 1972;39. 5034289

[27] Schenk RK Willenegger HR. [Histology of primary bone healing: modifications and limits of recovery of gaps in relation to extent of the defect (author’s transl)]. Unfallheilkunde. 1977;80:155–60. 867590

[28] Harris WH White RE Mccarthy JC. Bony ingrowth fixation of the acetabular component in Canine hip joint arthroplasty. Clin Orthop Relat Res. 1983:7–11. 6851344

[29] Lee KM Kim YI Park SB. Alveolar bone loss around lower incisors during surgical orthodontic treatment in mandibular prognathism. Angle Orthod. 2012;82:637–44. 22214389 10.2319/081711-526.1 PMC8845561

